# Supplementary material for: Pre-Chilling CGA Application Alleviates Chilling Injury in Tomato by Maintaining Photosynthetic Efficiency and Altering Phenylpropanoid Metabolism
Source: Plants (Basel). 2025 Jul 2;14(13):2026. doi: 10.3390/plants14132026 (PMC12251621; doi:10.3390/plants14132026)
Supplement: Supplementary file 1 [file plants-14-02026-s001.zip › plants-3680781-supplementary.pdf]

Pre-chilling application of chlorogenic acid can alleviate chilling injury in tomato by altering phenylpropanoid metabolism and maintaining photosynthetic efficiency

Yanmei Li [1a], Hanshuangfei Hu [2a], Qiang Guo[3], Luis A.J. Mur[4], Rui Guo[5*], Xiangnan Xu [1*]

1. Institute of Plant Nutrition and Environmental Resources, Beijing Academy of Agriculture and Forestry Sciences, No.9 Shuguanghuayuan Midroad, Haidian District, Beijing
2. Baosteel Engineering & Technology Group Co., Ltd, Shanghai, 201999, China.
3. Institute of Grassland, Flowers, and Ecology, Beijing Academy of Agriculture and Forestry Sciences, No.9 Shuguanghuayuan Midroad, Haidian District, Beijing, 100097, China.
4. Department of Life Sciences, Penglais Campus, Aberystwyth University, UK, SY23 3DA
5. Technical Centre for Soil, Agriculture and Rural Ecology and Environment, Ministry of Ecology and Environment, Beijing, 100012, China

SI. Text S1. The detailed half strength Hoagland nutrition solution recipe

KNO_3 - 253 ppm, KH_2PO_4 - 68 ppm, H_3BO_3 - 3.1 ppm, $CuSO_4 \cdot 5H_2O$ - 0.013ppm, $MnSO_4 \cdot H_2O$ - 8.5 ppm, KI - 0.42 ppm, NH_4NO_3 - 40 ppm, $MgSO_4$ - 120 ppm, $CoCl_2 \cdot 6H_2O$ - 0.013ppm, FeNaEDTA - 18.4 ppm, $NaMoO_4 \cdot 2H_2O$ - 0.13ppm, $ZnSO_4 \cdot 7H_2O$ - 4.3 ppm.

SI. Text S2. The product number of the Solarbio kits used in analysis

Malondialdehyde (MDA) - BC0025，Hydrogen peroxide (H_2O_2) - BC3595, Superoxide (O_2^-) - BC1295, Total hydroxyl radical-scavenging capacity (TOC) - BC1325, Total antioxidation capacity (T-AOC) - BC1315.

SI. Text S3. The detailed processes of transcriptome analysis

The total RNA in tomato leaves were firstly extracted using TRIzol® Reagent according the manufacturer's instructions. Then RNA quality was determined by 5300 Bioanalyser (Agilent Technologies Inc., CA, USA) and quantified using the Nano Drop ND-2000 (Thermo Fisher Scientific, MA, USA). The high-quality RNA samples (OD260 / 280 = 1.8 ~ 2.2, OD260 / 230 ≥ 2.0, RQN ≥ 6.5, 28S : 18S≥ 1.0, >1μg) were used to construct the sequencing library. The purification and reverse transcription of RNA, library construction and sequencing purification were performed at Shanghai Majorbio Bio-pharm Biotechnology Co., Ltd. (Shanghai, China) according to the manufacturer's instructions.

The tomato (*Solanum lycopersicum L.*) RNA-seq transcriptome library was prepared following Illumina® Stranded mRNA Prep Ligation (Illumina Inc., San Diego, CA, USA) using 1μg of total RNA. Shortly, messenger RNA was isolated according to polyA selection method by oligo (dT) beads and then fragmented by fragmentation buffer firstly. Secondly, the double-stranded cDNA was synthesized using a SuperScript double-stranded cDNA synthesis kit (Invitrogen, CA) with random hexamer primers. Then the synthesized cDNA was subjected to end-repair, phosphorylation and adapter addition according to library construction protocol. Libraries were size selected for cDNA target fragments of 300 bp on 2% Low Range Ultra Agarose followed by PCR amplified using Phusion DNA polymerase (NEB) for 15 PCR cycles. After the quantification by Qubit 4.0, the sequencing library was performed on DNBSEQ-T7 platform (PE150) using DNBSEQ-T7RS Reagent Kit (FCL PE150) version 3.0.

The raw paired end reads were trimmed and quality controlled by fastp [53] with default parameters. Then clean reads were separately aligned to reference genome with orientation mode using HISAT2 [34]

software. The mapped reads of each sample were assembled by StringTie [35] in a reference-based approach.

To identify DEGs (differential expression genes) between two different samples, the expression level of each transcript was calculated according to the transcripts per million reads (TPM) method. RSEM [36] was used to quantify gene abundances. Essentially, differential expression analysis was performed using the DESeq2 [54]. DEGs with $|\log2FC| \geqq 1$ and FDR $<$ 0.05 (DESeq2) were considered to be significantly different expressed genes. In addition, KEGG functional-enrichment analysis was performed to identify which DEGs were significantly enriched in KEGG terms and metabolic pathways at Bonferroni-corrected p-value < 0.05 compared with the whole-transcriptome background. The KEGG pathway analysis were carried out by Goatools and Python scipy software, respectively.

SI. Text S4. The detailed processes of metabonomic analysis

For tomato leaf, 0.5 g solid sample was weighed into 10 mL grinding tube and added with 5 mL of 0.02 mg mL^{-1} internal standard (L-2-chloro-phenylalanine, dissolved in 80% methanol solution. A steel ball and 2 mL of chloroform were further added to the tube, and the sample was frozen and ground at 50 Hz for 6 min, ultrasonicated at low temperature for 30 minutes and left to stand for 30 min (-20 °C), then centrifuged at 13000 rfc for 15 min (4 °C). The supernatant was transferred into a glass derivative vial and blown dry in steady nitrogen stream. Then, 80 μL of methoxypyridine hydrochloride solution (15 mg mL^{-1}) was added to the glass-derived vial. After vortex shaking for 2 min, the oximation reaction was carried out in a shaking incubator for 90 min (37 °C). The mixed solution was added with 80 μL BSTFA derivatization reagent (containing 1% TMCS), vortexed and shaken for 2 min, and then the reaction was carried out for 60 min (70 °C). Eventually, the samples were removed and left at room temperature for 30 minutes, pending analysis.

The GC-MS analysis was performed by an Agilent 8890B gas chromatography coupled with an Agilent 5977B mass selective detector at Majorbio Bio-Pharm Technology Co. Ltd. (Shanghai, China). The instrument is equipped with an inert electron impact (EI) ionization source with an ionization voltage of 70 eV (Agilent Technologies Inc., CA, USA). The samples were separated with a DB-5MS (40m × 0.25 mm × 0.25 μm) capillary column, using 99.999% helium as a carrier gas at a constant flow rate (1 mL min^{-1}). The GC column temperature was programmed to hold at 60 °C for 30 s and rise to 310 °C at a rate of 8 °C per minute. The injection volume of samples was 1 μL and introduced in splitting mode (15:1) with the inlet temperature of 260 °C. The mass spectrometry conditions are as follows: The ion sources temperature was 230 °C and the quadrupole temperature was 150 °C. The scanning mode is

full scan mode, the quality scanning range is m z^{-1} 50-500, and the scanning frequency is 3.2 scan s^{-1}.

A quality control sample (QC) was prepared to evaluate the system stability. The QC samples were made by mixing all test samples and were treated in the same way as formal samples. During the formal testing, one QC sample was inserted every 5-15 samples. The raw data obtained from mass spectrometer detection of GC-MS was preprocessed by MassHunter workstation Quantitative Analysis (version v10.0.707.0) software, and a three-dimensional data matrix in CSV format was exported. At the same time, the metabolites were identified by searching database MS-DIAL (version 2021). Then, the data matrix obtained was uploaded to the Majorbio cloud platform (https://cloud.majorbio.com) for further analysis.

Firstly, the data matrix was pre-processed that at least 80% of the metabolic features detected in any set of samples were retained. The samples with a metabolite level below the quantification lower limit was screened out. To reduce the errors caused by sample preparation and instrument instability, the response intensities of the sample mass spectrometry peaks were normalized using the sum normalization method to obtain the normalized data matrix. Meanwhile, the variables of QC samples with relative standard deviation (RSD) > 30% were excluded and log10 logarithmicized to obtain the final data matrix for subsequent analysis.

Then, the R package "ropls" (Version 1.6.2) was used to perform principal component analysis (PCA) and orthogonal least partial squares discriminant analysis (OPLS-DA), and 7-cycle interactive validation evaluating the stability of the model. The metabolites with variable importance in the projection (VIP) > 1 and $p < 0.05$ were determined as significantly different metabolites based on the VIP obtained by the OPLS-DA model and the p - value generated by Student's T test [33].

SI. Text S5. The detailed figure description of Figure 2, Figure 3 and Figure 4.

The Figure 2(a) was the Venn analysis of the differentially expressed genes (DEGs) found in LL vs CK and LL-CGA vs CK. The LL vs CK and LL-CGA vs CK shared 986 same DEGs, occupying 14.51% of the whole DEGs pool from both comparisons, and the number of DEGs solely found in LL vs CK or LL-CGA vs CK were 5081 and 726, occupying 74.80% and 10.69% of the whole DEGs pool from both comparisons, respectively.

The Figure 2(b) was the PCA analysis for the tomato leaf transcriptome results. The X-axis was the principal component 1, which explained 58% of the total sample variance, and the Y-axis was the principal component 2, which explained 14% of the total sample variance. The data points representing CK, LL and LL-CGA clustered into three groups, the confidence eclipse of CK separated from the eclipses of LL and LL-CGA, whilst the separation between LL and LL-CGA was not distinctive.

For better explaining the difference of the plant performance between LL and LL-CGA, the DEGs between LL-CGA and LL were also analyzed. After screening for the significant DEGs, the genes were annotated based on KEGG pathways. As shown in Figure 3(a) - 3(d), the LL mainly influenced the cell energy metabolism, RNA translation and lipid metabolism, which had downregulated 14 genes regulated energy metabolism, 6 genes regulated RNA translation and three genes regulated lipid metabolism, respectively; while on the basis of LL, the LL-CGA mainly influenced the cell energy metabolism, which had upregulated 16 genes regulated energy metabolism. The KEGG enrichment analysis bubble charts also showed that the energy metabolism were the main regulation pathways through which the LL and LL-CGA impacted the plant performance. As the Figure 3(e) and 3(f) shown, the significant DEGs from LL vs CK and LL-CGA vs LL mainly enriched into photosynthesis pathway and oxidative

phosphorylation pathway, which were the two most important energy metabolism pathways in plant growth.

For better exploring the mechanism of how the CGA relieved tomato plants from cold stress, the weighted correlation network analysis (WGCNA) was performed to all the DEGs found from LL-CGA vs LL, and the results were shown in Figure 4(a) and Figure 4(b).

The Figure 4(b) showed detailed correlation levels between the individual genes and phenotype traits, and the more reddish color indicating higher positive correlation and the more greenish color indicating higher negative correlation. In yellow and green modules, genes were positively correlated with shoot fresh mass (SF), leaf TOC, T-AOC, Fv/Fm, Eto/RC, shoot N, root Ca and root Zn, but were negatively correlated with leaf MDA content, H_2O_2, O_2^-, DIo/RC, root N, shoot Ca, root Fe, Shoot Mn, root Mn and shoot Zn and shoot Fe; in module blue, majority of the genes were generally negatively correlated with shoot fresh mass, leaf MDA content, H_2O_2, O_2^-, TOC, T-AOC, Fv/Fm, shoot N, root Ca, shoot Fe, shoot Mn, root Mn, shoot Zn and root Zn, but were generally positively correlated with DIo/RC, ETo/RC, root N, shoot Ca and root Fe, whilst there are minority of genes showed positive correlation with SF, TOC, T-AOC, Fv/Fm, ETo/RC, root N, root Fe and root Zn, and simultaneously showed negatively correlation with leaf MDA, H_2O_2, O_2^-, DIo/RC, shoot Ca, shoot Fe, shoot Mn, root Mn and shoot Zn; in grey module, the only one gene show similar pattern to blue module in its correlation with these traits analyzed; the genes belonging to turquoise module generally showed negative correlation with SF, TOC, T-AOC, Fv/Fm, Eto/RC, shoot N, root Ca and root Zn, but showed positive correlation with leaf MDA, H_2O_2, O_2^-, DIo/RC, root N, shoot Ca, root Fe, shoot Mn, root Mn and shoot Zn; in red module the genes generally showed negative correlation with Eto/RC, root N, root Fe, and showed

positive correlation with leaf MDA, TOC, T-AOC and Fv/Fm, shoot N, root Ca, shoot Fe, shoot Mn, root Mn and shoot Zn, whilst other traits do not show a consistently clear pattern in their correlation with the genes in red module; interestingly, the genes in brown module showed divergent pattern in their correlation with the traits analyzed, that about half genes have a same pattern as the genes in turquoise module do, but another half show the dame pattern as the red module does.

SI. Figure S1. The OPLS-DA results for the differential accumulated metabolites (DAM) analysis undertaken between LL and CK (a) as well as LL-CGA and LL (b).

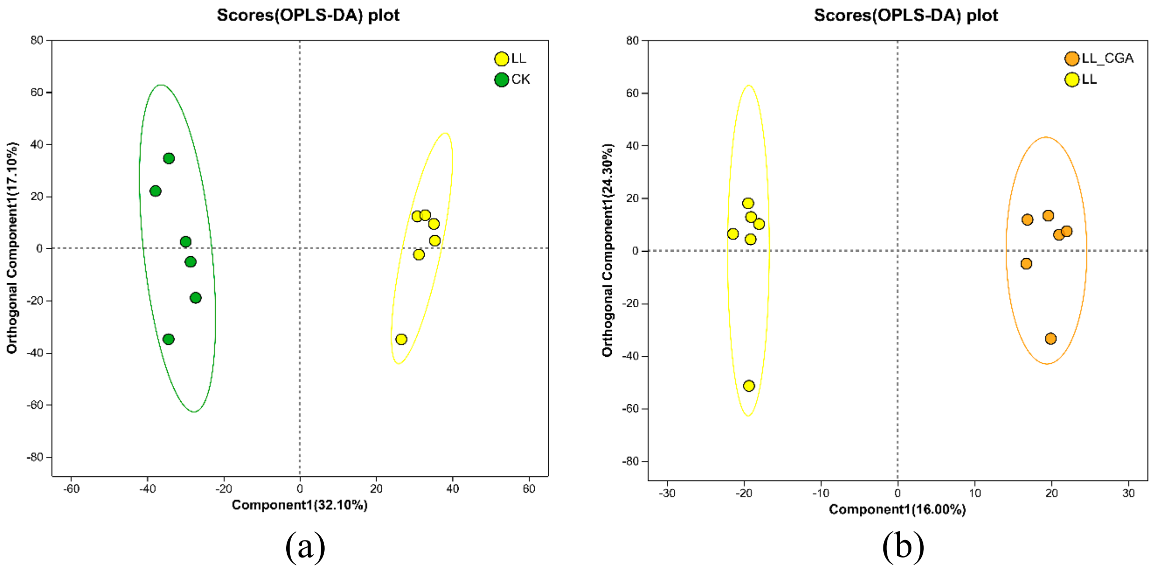

SI. Figure S2. The individual results of significant DEMs relevant to phytohormone synthesis defined for LL vs CK.

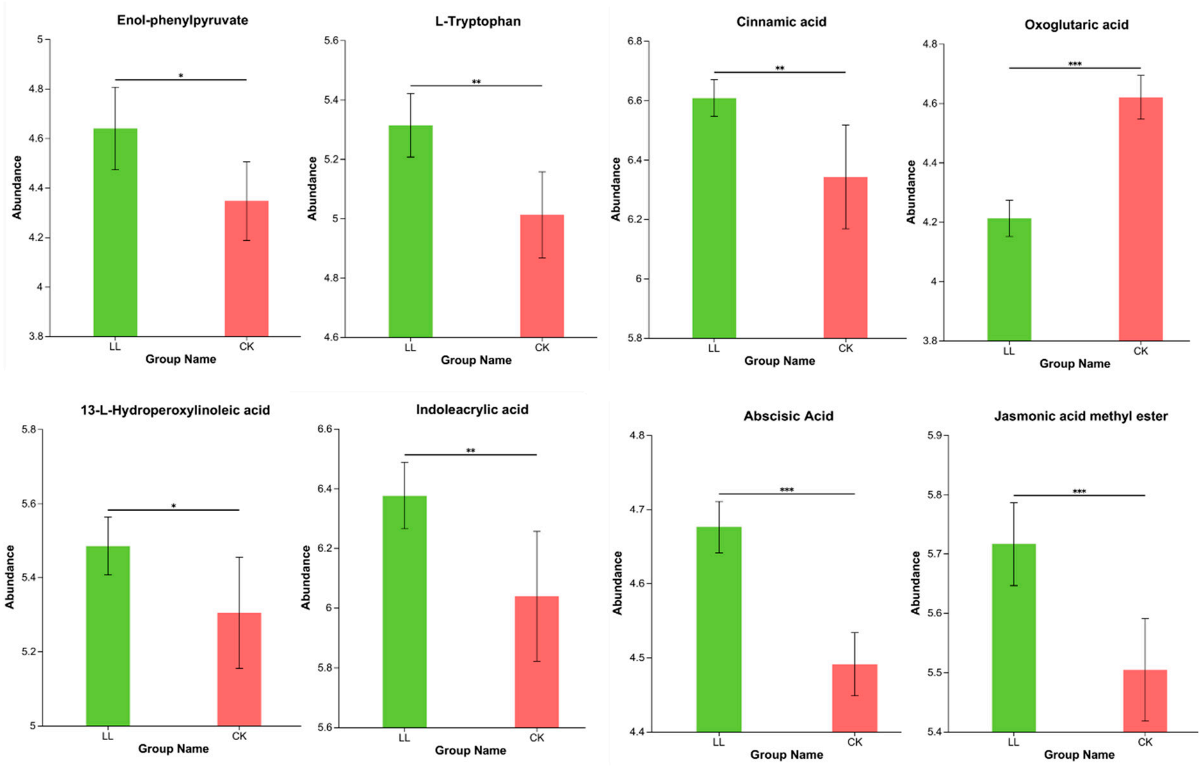

SI. Figure S3. The individual results of significant DEMs relevant to phytohormone synthesis defined for LL-CGA vs LL.

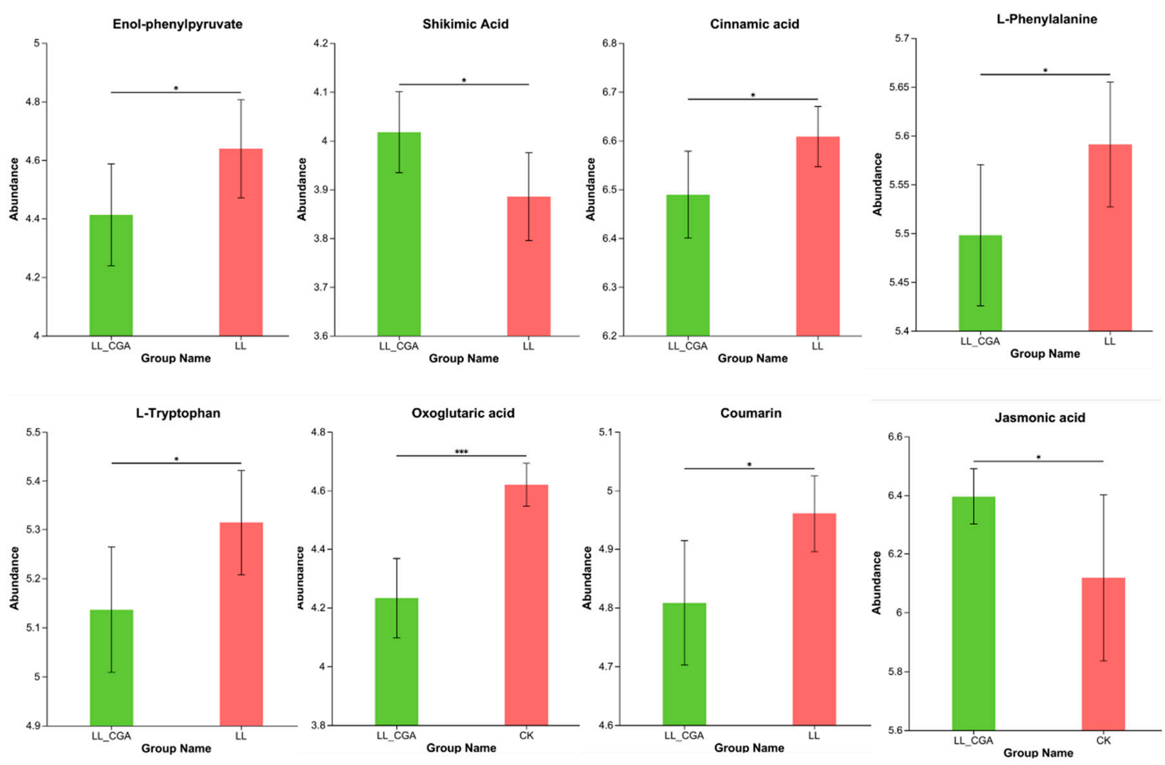

SI. Figure S4. The photo result showing plant performance from pre-trial

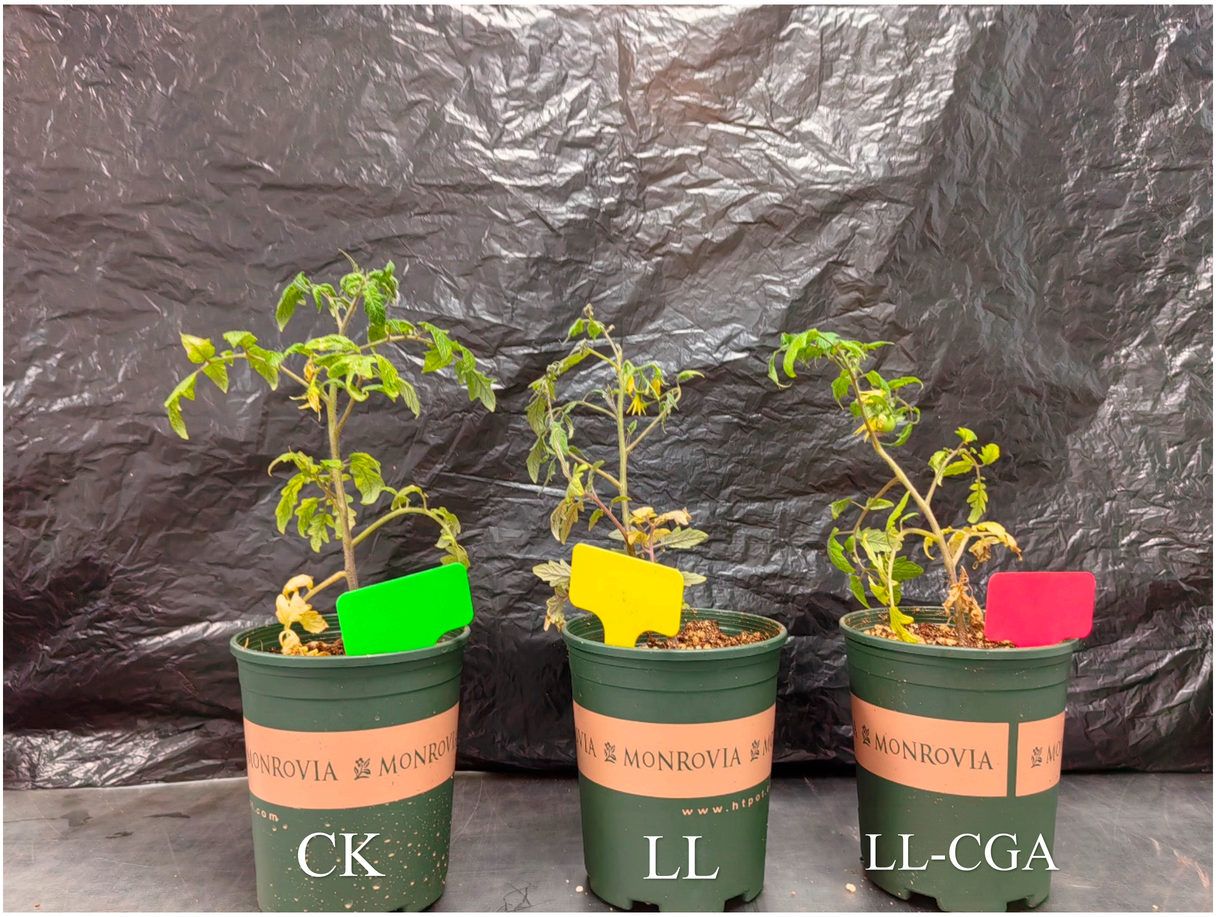

SI. Table S1. Shoot sulfur concentration of the tomato plant after experiment

Treatment	S<br>g kg^{-1}
CK	8.47±0.48
LL	8.32±0.26
LL-CGA	8.90±0.37
